# Correlating Time Series Signals and Event Logs in Embedded Systems

**DOI:** 10.3390/s21217128

**Published:** 2021-10-27

**Authors:** Kazimierz Krosman, Janusz Sosnowski

**Affiliations:** Institute of Computer Science, Warsaw University of Technology, 00-665 Warsaw, Poland; krosman.kazimierz@gmail.com

**Keywords:** signal processing, embedded systems, data synchronisation issues, device monitoring, time series analysis

## Abstract

In many embedded systems, we face the problem of correlating signals characterising device operation (e.g., performance parameters, anomalies) with events describing internal device activities. This leads to the investigation of two types of data: time series, representing signal periodic samples in a background of noise, and sporadic event logs. The correlation process must take into account clock inconsistencies between the data acquisition and monitored devices, which provide time series signals and event logs, respectively. The idea of the presented solution is to classify event logs based on the introduced similarity metric and deriving their distribution in time. The identified event log sequences are matched with time intervals corresponding to specified sample patterns (objects) in the registered signal time series. The matching (correlation) process involves iterative time offset adjustment. The paper presents original algorithms to investigate correlation problems using the object-oriented data models corresponding to two monitoring sources. The effectiveness of this approach has been verified in power consumption analysis using real data collected from the developed Holter device. It is quite universal and can be easily adapted to other device optimisation problems.

## 1. Introduction

Various sensors are widely used in diverse domains and the collected data need quite sophisticated processing for cognitive or reactive activities. This triggered the development of tiny and low-cost devices installed in the field. They are based on microcontrollers including a system on chip with memory and communication circuitry (embedded systems, IoT—Internet of Things devices, SCADA—supervisory control and data acquisition nodes). The available functional block resources are limited, which is opposed to increasing demands of advanced data processing and interaction with the environment. Hence, in developing practical application systems, we face the problem of optimizing data processing algorithms, device resource usage, dependability, performance, and power consumption. An important issue is testing and validation of relevant device prototypes in simulation or production environments. In practice, this process needs efficient real-time monitoring of the device’s operation. It involves observation of selected physical signals and device and environment states ([1,2] and references therein). From an analytical point of view, this leads to correlation analysis of time series depicting considered signal states and relevant device state/event logs.

Most research papers on signal monitoring and analysis deal with time series decomposition, classification, prediction, and characteristic features; some publications are commented on and referred to in Section 2 (e.g., [3,4,5]). More advanced analysis of practical problems (e.g., device optimisation, anomaly detection) involves the need for also considering other data sources, e.g., event logs ([6,7] and references therein). In the case of external monitoring of signals (typically, with special data acquisition equipment), we face the problem of time correlation of the derived time series (collected data samples) and other logs generated by the monitored system, usually not synchronised. This problem is neglected in the literature, and we showed its practical importance [2] while analysing time series features at a higher observation level involving data sample aggregation into time series objects (e.g., pulses, characteristic sample sequences). 

As shown in Section 2, classical correlation and synchronisation schemes are not satisfactory due to the limited capabilities of interactions between data acquisition and embedded monitored devices. Hence, we have developed an original solution which matches device logs with pointed object instances in time series. It takes into account clock offset fluctuation and log contextual factors. The introduced object-oriented data models for time series and event logs facilitated constructing efficient algorithms adapted to their features. This approach has been verified while developing commercial Holter devices. It can be used in a wide scope of other problems, especially those within embedded and IoT frameworks.

The rest of the paper is organised as follows. Section 2 gives a background of the considered problem in the context of the related literature. Section 3 outlines data models and the concept of the proposed analysis. Section 4 presents developed algorithms used to correlate time series data with event logs. A practical example of the analysis is presented in Section 5. Section 6 and Section 7 discuss and conclude our research, respectively.

## 2. Problem Statement and Related Works

Many electronic devices are used to interact with real physical processes and an external environment. They involve effective data processing resources and signal sensors. Analysing the operation of such devices needs tracing various signals and events. For this purpose, special external data acquisition tools and internal device monitoring mechanisms are needed. They provide two classes of data: time series (TS) related to diverse observed signals and events correlated with internal or external state/behaviour changes. In the literature, there are a lot of studies focused on either time series or event logs separately. 

Within time series analysis, we distinguish four research goals: (i) TS decomposition [3,8], which it involves deriving trend, season, noise and other specified components; (ii) TS classification [9,10,11], based on comparing time series with each other and finding similarities using diverse metrics; (iii) deriving characteristic and anomalous features [12,13,14]; and (iv) predicting future behaviour [3,4,15] by finding data patterns for the near future based on the previous/historical ones. They are considered as general or application-targeted problems. Quite sophisticated time series coherence studies, based on LSCWA (Least-Squares Cross-Wavelet Analyses) and XWT (Cross-Wavelet Transform), are presented in [16,17], respectively. These decomposition methods provide time lag information between components (used in satellite image and geological time series studies). In [18], the authors give a comprehensive survey of the main time series decomposition strategies (including deterministic and stochastic features) and their relative performances in different application domains. A multichannel signal decomposition approach is presented in [19] and illustrated in the analysis of real-life EEG and vibration signals. Event log analyses are mostly targeted at classification problems and detection of anomalous situations, e.g., the appearance of suspicious events or their sequences ([7,20] and references therein), grouping logs into event sequences (workflows) and log reduction (compression) [21]. This is supported with log parsing algorithms [6,22].

Typically, sensors generate data samples at regular intervals, and they can be treated as time series (TS). TS represent some variable values of observed device or environment behaviour, e.g., temperature and memory and processor usage. Logged events carry useful information on the device activities, their context, device state changes, etc. In practice, TS and events are collected separately for different purposes, which creates some difficulty in the correlation analysis. Correlating time series with event logs provides the additional context of the underlying device activities and anomalies.

The multitude of collected data in monitoring processes arises the problem of their correlation ranges. This can be studied from global or local perspectives with fine- or coarse-grained views considering diverse individual or aggregated features, respectively. For example, we can trace factors impacting the total power consumption of the device or its relevant functional blocks, respectively. In many papers, authors study correlations between diverse signals described by relevant TS [23,24]. In classical approaches, Pearson correlation metrics are used. This is also extended for time series classification, e.g., based on extracting mean and trend features of TS and finding similarity metrics for classification [11]. Whole-series and feature-based algorithms are used here. The latter transforms TS into the representation of feature vectors. The problem of aligning several TS in a common time scale is presented in [25]. Abrupt signal change correlations in multivariate TS are studied in [26]. Time scale-dependent correlations and decomposition of TS are considered in [27]. Event log correlation is focused on finding co-occurrences of different types of events or alert reports [20,28]. In [24], faults are identified by a sequence of events, based only on their temporal arrival pattern. The problem of correlating TS with collected events is rarely encountered in the literature. 

In [29], correlation between an event sequence and time series is targeted at incident diagnosis in the system. Temporal order and monotonic effect of dependencies are examined here. This results in tracing event occurrence corresponding to a significant value change in a time series. In [30], the authors present an interactive graphical tool facilitating finding correlated events with specified points in time series plots. They focus on a single event correlation and assume a consistent time scale for both types of data. Simultaneous exploration of event and time series data is performed in an interactive way by deriving events related to a marked point on time series sample plot. This is cumbersome in case of long time series, moreover, it lacks an aggregated view of the collected data. The problem of discovering correlations of TS changes with single events is outlined in [31], as it assumes consistent time scales. In [32], time series specifying meteorological parameters (humidity, temperature, bulb, wind direction, etc.) recorded in hourly intervals are correlated with critical events (heavy rains, flooding, hurricanes, etc.). The dynamics of these data is relatively low as compared with monitoring capabilities; moreover, the set of considered events is limited, so timing problems do not appear here. In the case of many embedded systems, we face the problem of fine-grained time series and rich event logs needing frequent observations. Here, data acquisition and device time scale consistency/fluctuation problems must be considered in the analysis, which is neglected in the literature.

The interpretation of acquired TS data from the monitoring system needs referring to event logs registered by the monitored device. Dealing with data provided by different sources requires a common notion of time. Hence, the problem of data synchronisation arises. In the literature, various synchronisation algorithms have been proposed, especially for distributed and IoT systems. They are based on exchanging synchronisation messages [33] or on time compensation schemes [34]. Unfortunately, using these approaches in external device monitoring is not satisfactory due to limited capabilities of accurate data acquisition systems. We faced this problem while developing some embedded and IoT devices [2]. The monitored embedded devices quite often do not provide hardware/software synchronisation capabilities or do not accept the impact of additional synchronisation processes on their operation. Moreover, time fluctuations in event log registration may appear due to device software interacting processes. In this situation, we rely on combined time and context-oriented correlations. 

As opposed to classical approaches dealing with TS at sample level, we consider TS objects aggregating samples, e.g., pulses, series of pulses, snippets, states, and state sequences. We showed the usefulness of aggregating TS samples into objects in [2,28] while analysing operations of embedded and complex computer systems, respectively. The presented studies in subsequent sections of this paper correlate not individual events but event sequences. In the case of similar TS object instances (e.g., similar pulses), the correlated sequences of events may differ; therefore, it is important to detect the most crucial events with high correlation probability. This results in a quite complex algorithm, integrating the following problems:

- Adapting time scales of TS and event logs.
- Correlating event logs with specified TS objects, including their sequences (instances).
- Selecting dominating events over the studied object instance series.

The developed correlation approach has been verified on real data collected from Holter device. Nevertheless, it can be easily adapted to other projects consistent with the quite universal data model presented in Section 3.

## 3. Data Models

The developed correlation analysis targets time series (TS) and event log (EL) data sets. Time series TS = {s_1_, s_2_, …, s_r_} is composed of data sample values (s_i_, 1 ≤ I ≤ r) collected with sampling time T defined by the local clock of the monitoring (data acquisition) device. Within TS, we can distinguish objects defined as subsets of samples with specified properties, e.g., higher average values within a predefined window time and pulses of specified shapes. Decomposing TS into objects can be performed manually or using special algorithms targeted at pulses or more complex object features, as illustrated in our previous papers [2,28], respectively. Subsequent object instances can appear in diverse moments of the TS time scale. In the correlation analysis, we focus only on the time intervals relevant to subsequent instances of the considered objects, defined as the set of time intervals OI = {(t_1_, t_2_), (t_3_, t_4_), …, (t_k_, t_k+1_)}. Each element *z_i_* of the set OI is a pair of timestamps t_i_, t_i+1_ specifying time range of the *i*-th object; we also use notations *z_i_*(1) and *z_i_*(2) for these initial and closing timestamps of the *z_i_* interval, respectively. 

Event logs are collected within the monitored device and are stored as a set of log records EL = {E_1_, E_2_, ……E_v_}. Each event record comprises a relevant timestamp and a textual message composed of words and terminated by the new line character. The timestamp is generated in relevance to the local clock of the monitored device—it is independent (not synchronised) from the monitoring device clock; however, the accuracy and fluctuation ranges of these clocks are known. Some specific data fields can be distinguished within the textual message depending on the assumed logging scheme, e.g., we may have the tag/service/module of the program that generated the log event. 

The considered correlation problem is the optimal matching of the TS object intervals OI with relevant events within the EL set. This process is based on deriving relations of OI and EL elements within time scales linked with TS and EL data repositories. Developing correlation algorithms, we introduce some data entities and notations. Most of them specify EL record properties. The data model specifications use classical logical and mathematical notations: (a) $\cup_{x}Y_{x’}$ union of sets Y_x_ specified by index condition x; (b) $\sum_{x}Y\left(x\right)$, algebraic sum of elements Y(x) specified by index condition x; (c) $\forall x,\;Y\left(x\right)$, universal quantifier, denotes that property Y(x) is satisfied for every x; and (d) $\exists x,Y\left(x\right)$, existential quantifier, denotes that there is x for which property Y(x) is satisfied. Operation A∩B denotes the conjunction of sets A and B. Relation $a_{j}\in A$ denotes that element a_j_ is included in set A. 

The basic entity in logs is word $w_{i}\in \bar{W}$ defined as a sequence of characters (e.g., in ASCI code) separated with specific symbols, e.g., space _, −, [,], (,), +. For the performed analysis, it is reasonable to classify words. Based on the experience with embedded system logs, we introduce the following word types: (1) *l-word*, any sequence of characters starting with a letter character; (2) *d-word*, composed only of digital characters 0–9 but different from types 3 and 4; (3) *PID*, specifies the instance of the process generating the relevant log; (4) *source*, program service or module responsible for the log generation; and (5) t_L_, log *timestamp*. This classification is supported with log parsing. The *classified word* is defined as a pair $cw=\left(w_{i},\;m_{j}\right),\;\;where$ $w_{i}\in \bar{W}\;and\;m_{j}\in \bar{M}$, $\bar{M}=\left\{l-word,\;d-word,\;pid,\;source,\;timestamp\right\}$. We use also notations *cw*(1*)* and *cw(*2*)* to denote the word and its type (category), respectively. 

Each log record/entry L can be represented as a bag of words B_L_ defined as the set of classified words. Function TS(B_L_) gives the timestamp of this log. Function BW(*cw_i_*) assigns a numerical value (weight) to the classified word (*cw_i_*) depending on its type, i.e., *cw_i_*(2) value. For types *l-word*, *d-word, pid, source*, and *timestamp* we assumed these values as: 1.0, 0, 3.0, 2.0, and 0.0, respectively. Comparing two bags of words, *B_a_* and *B_b_*, we use the similarity function defined as follows:

$$SIMILARITY\left(B_{a},\;B_{b}\right)=\frac{\sum_{cw_{i}\in B_{a}\cap B_{b}}BW\left(cw_{i}\right)}{\max\left\{\sum_{cw_{j}\in B_{a}}BW\left(cw_{j}\right),\sum_{cw_{k}\in B_{b}}BW\left(cw_{k}\right)\right\}}$$

We introduce a set of similar bags of words (SBSx) which is derived with a special algorithm specified in Section 4. For each pair of bags of words *B_a_* and *B_b_* in SBSx, we have SIMILARITY(*B_a_*, *B_b_*) ≥ *eps_s*, where *eps_s* is the assumed minimal similarity threshold. SBSx comprises all similar bags of words derived for the whole log set EL. Each bag of words in EL is assigned to a single similarity set SBSx. 

*Event sequence* S is defined as an ordered (according to timestamps) set of similar bags of words satisfying the following condition:

$$S\subseteq \;SBS_{x},\;\forall \;B_{j}\in SBS_{x}/S,\forall \;B_{k}\in S,|TS\left(B_{j}\right)-TS(B_{k})|>eps\_tolerance_{}$$

where *eps_tolerance* is the parameter partitioning set SBSx. Elements of event sequence satisfy relation: $TS\left(S\left(1\right)\right)\leq TS\left(S\left(2\right)\right)\leq TS\left(S\left(3\right)\right)\leq \ldots \leq TS\left(S\left(N\right)\right)$, where *N* is the cardinality of sequence *S* and *TS(S_i_)* is the relevant timestamp of the last element of sequence *S_i_*. In this way, we can partition set *SBS_x_* into subsequent sequences constituting an ordered set *D_x_* = {*S*_1_, *S*_2_, …, *S_Mx_*}, which creates a series. We denote the set of all sequences of *SBS_x_* as $\bar{S\left(SBS_{x}\right)}$. The intuition of this partitioning is to derive similar event groups close in time (potentially possible to correlate with TS object time intervals) and separated from other groups by a longer time distance > *eps_tolerance,* possibly related to another TS object instantiation. 

We define the set *IS(SBSi)* of intersequence gaps, i.e., sets of timestamp differences between subsequent elements of *D_i_* series. We denote the timestamp of the first bag of words of the *j*-th sequence in *D_i_* as *TS_DB_(D_i_(j))* and the last one as *S_DE_(D_i_(j))*. Hence, we have:

$$IS\left(SBS_{i}\right)=\left\{TS_{DB}\left(D_{i}\left(2\right)\right)-TS_{DE}\left(D_{i}\left(1\right)\right);\;TS_{DB}\left(D_{i}\left(3\right)\right)-TS_{DE}\left(D_{i}\left(2\right)\right);\ldots ;TS_{DB}\left(D_{i}\left(N\right)\right)-TS_{DE}\left(D_{i}\left(N-1\right)\right)\right\}$$

Taking into account that in many embedded or IoT systems, we can observe a background of diverse secondary events usually performed in a periodic way, we define noise set of bags of words *ZW_i_* satisfying the following condition:

$$\frac{\left|avg\left(IS\left(SBS_{i}\right)\right)-med\left(IS\left(SBS_{i}\right)\right)\right|}{avg\left(IS\left(SBS_{i}\right)\right)}<noise\_0_{}\;AND\;\frac{std\left(IS\left(SBS_{i}\right)\right)}{avg\left(IS\left(SBS_{i}\right)\right)}<noise\_1$$

where *noise_0* and *noise_1* are predefined parameters of the developed algorithm, and *avg*, *med,* and *std* denote average, median, and standard deviation values calculated over intersequence gap sets, respectively. The correlation analysis involves the target bag of words which satisfies the same conditions with replaced < relations into ≥ (described in Section 4). 

In the correlation analysis, we search for the set of matched object intervals (*OI*) with event sequences denoted as *RES* set, which is generated with a set of algorithms specified in Section 4. Set *RES* is defined as follows (*z*(1) and *z*(2) denote initial and closing timestamps of the interval z, respectively):

$$RES\left(SBS_{x},k\right)=\{S\subseteq \bar{S\left(SBS_{x}\right)}:\;\forall z\in OI,\exists B_{j}\in S,\;TS(B_{j}\left(1\right))+k\geq \;z\left(1\right)\;AND\;TS(B_{j}\left(N_{Bj}\right))+k\leq z\left(2\right)\}\;RES\left(k\right)=\underset{SBS_{x}\in \bar{SBS}}{\cup }RES\left(SBS_{x},k\right)$$

For the given set of similar bags of words *SBS_x_* and the offset value *k,* we create a set that contains only selected sequences from the set *SBSx*. The sequence initial and final timestamps with the added offset *k* must not exceed time limits defined by the considered interval *z* in OI. Iterating through all the sets of similar bags of words and joining all *RES*(*SBSx*, *k*) sets, we create set *RES*(*k*). *RES*(*k*) is the set of all sequences that can fit inside the instances of time series object intervals by adding the *k* value to their timestamps. The searched matching offset value k generates the *RES*(*k*) set with the greatest cardinality. 

The developed algorithms (Section 4) closely relate to the introduced data models. For better understanding, data models and their analysis are illustrated in Section 5 in relevance to time series objects and event logs. The included Appendix A summarises used acronyms to facilitate tracing the presented considerations.

## 4. Specification of Algorithms

In the developed correlation analysis, we distinguish two phases: (1) extracting sub-sequences of event logs that can correspond to the object intervals (see Section 3), and (2) finding time offset assuring best matching of the object and event log timing. The general idea of the correlation scheme is presented in Algorithm 1, which uses various functions defined subsequently in the text. Algorithms and functions are specified in a pseudocode partially based on object-oriented programming. It is consistent with the introduced data model (Section 3) and it was also used in our previous paper [2] targeted at time series decomposition.

The configuration parameters of the algorithms are: (1) *word_weights*, (2) *eps_s*, (3) *eps_time*, (4) noise parameters: *noise_0* and *noise_1*, (5) *time_increment*, and (6) *eps_tolerance.* The values of parameters (1) and (2) are crucial for bags of words clustering into sets of similar bags. The *word_weights* influence the similarity metrics results. The weights for word types such as *PID* or *source* should be greater because it is common that similar logs or even identical logs are generated by a single service. Moreover, if the specific log format is known, adjusting weight values can increase the accuracy of detecting similar bags. Parameter (2) allows us to discriminate similar bags from others. It can take values between 0.0 and 1.0. The lower it is, the more false positive cases it generates. The specific value depends on a variety of logs and can be selected experimentally by analysing similar bags of words sets. Parameter (3) impacts the number and all statistical features of generated sequences. For higher values, the single sequence can be potentially longer (in time and the number of bags). If the value is too high, the algorithm generates one sequence for each similar set of bags. The proper value is related to the time characteristics of the interval objects, especially the average time of the single object instance and the maximal or minimal time interval between consecutive instances. The noise parameters are used to detect periodical sequences. The value for both *noise_0* and *noise_1* is set to 0.2 but can be increased for periodic sequences with some jitter. Parameter (5), *time_increment* value, influences the number of matching candidates and should be dependent on event log density. Reducing this value increases the accuracy of the algorithm up to some level. For example, if the shortest time interval between two event logs is 5 s, setting *time_increment* to 5 s and parameter (6) *eps_tolerance* to about 2.5 s will generate the best results in the context of accuracy. Moreover, for lower values of parameter (5), the algorithm execution time increases. Increasing or decreasing parameter (6) can increase or decrease the number of the final set of candidates. The assumed values should not exceed the value of parameter (5). Most of parameters can be set experimentally once for specified event logs and then the algorithm can process other input data with similar format and timing characteristics.

The presented algorithms use pseudocodes based on C/C++/Pascal notation with bolded keywords. The function keyword starts the function definition that can be identified as a single procedure of the algorithm. The **foreach** logic structure is used to create loops. The **foreach** loop iterates through all elements in the collection. The collection name is inside the **foreach** parameter section specified in brackets. Instructions inside the loop (between keywords ***do*** and ***end***) are executed the same number of times as the number of elements in the container. Instructions executed in successive iterations can use successive elements of the container. The conditional instructions are consistent with classical **If** statements. All presented algorithms use an object-oriented approach. Complex data structures such as lists or objects that aggregate other data types are presented as objects of the specified class, and object instances are created with the **new** operation. The objects provide properties and methods (functions). The property can refer to an object internal collection. Referencing to object properties and methods is denoted with dot operator (.). Construction *object.method(arguments)* invokes some method (function) on the object that can return some data type of the object or change internal state of the object. Most used names of objects and methods are self-explanatory, others are additionally commented. For example, *filtered_sequences.add_sequence(s)* denotes adding sequence *s* (with the specified method/function) to the object *filtered_sequences*. 

The inputs to Algorithm 1 are two sets: *log_text* and *intervals* equivalent to specifications EL and OI in Section 3, respectively. Function *create_bags_of_logs* returns a list of a bag of words corresponding to considered logs. It partitions log records into words and generates objects of a bag of words as classified words (i.e., pairs word and its type—Section 3). For example, log: “R11-08 07:05:38.657 D/AT (134): AT > AT + CCWA = 1” is transformed into the equivalent bag of words: <08 07:05:38.657, *timestamp*>, <AT, *source*>, <134, *pid*>, <AT, l*-word*>, <CCWA, *l-word* >, < 1, *d-word*> (R11 and D/ denote real time clock and debug level of logging, respectively). The created bag of words list is used by function *cluster_into_ssb* which provides a classified similar bag of words (using similarity metric specified in Section 3). Each cluster comprising similar bags of words (*SBS_x_*) is partitioned into sequences. A single *sequences* object is composed of one or more consecutive (in time) sequences. Objects *sequences* and *intervals* are the arguments of procedure *candidates_from_one_sequences*. It returns candidates for matching object intervals with a single object of sequences. This is performed via the iterative checking of the matching result or subsequent time corrections with *offset* value. Candidates are aggregated in the list *candidates*. This list is used by the find*_best_matching* procedure, which returns the *candidates* list of candidates with a common offset (with tolerance defined by *eps_tolerance* parameter) that assures the maximal number of matched candidates. This list and the adjusted offset value are the result of the algorithm, which allows us to investigate and filter text logs matched with TS objects (corresponding to *RES*(*SBS_x_*,*k*) set defined in Section 3).
**Algorithm 1:** Data correlation.**Input**: *log_text-* the log records in text format, objects- the list of TS object intervals **Output:** logs in format bags of words matched with intervals 1: **function** match_events_with_logs (*log_text, intervals*) 2: logs_as_bags = *create_bags_of_logs(log_text)* 3: ssb = *cluster_into_ssb(logs_as_bags)* 4: candidates = **new** list 5: **foreach** (similar_bags **in** ssb) **do** 6: sequences = create_sequences(similar_bags) 7: candidates.add(candidates_from_one_sequences(sequences, objects)) 8: **end foreach** 9: matched_logs = find_best_matching(candidates) 10: **return** matched_logs 11: **end function**

Function create*_bags_of_logs(log_text)* invoked in line 2 of Algorithm 1 is relatively simple, so we skip the relevant pseudocode. It analyses log entry texts, performs tokenisation to identify words, and attributes appropriate word class (*l-word, d-word, PID, source, timestamp*) taking into account word contents and context resulting from assumed log formats. The result of this processing is the list *bags* with elements corresponding to subsequent log records. Each element comprises a relevant bag of words and the normalised timestamp. Timestamp normalisation is calculated by taking the timestamp of the first bag of words (corresponding to the first log record) and subtracting it from the timestamps of subsequent bags of words.

Algorithm 2 partitions bags of words into sets of similar bags of words (compare SBS in Section 3). In the first step, the list of bags of words is copied to a supplementary object *unmatched_bags*. In the *while* loop (line 4), a new element of the object (bag of words) is taken with the *take_first* method. It is used as a seed for the new cluster created with procedure *create_similar_bags_list* (Algorithm 3). This procedure searches the list *unmatched_bags* to find bags of words similar to at least one cluster element. Two bags of words *a* and *b* are similar if the similarity metric (defined in Section 3 and provided by function *similarity*(*b, a)*) is higher than the specified threshold by parameter *eps_s*. The added new element to the created cluster *similar_bags* is removed from the collection *unmatched_bags*. After finding all bags of words similar to the currently created cluster, this cluster is added as a list to the set *ssb*. Having assigned all bags of words to appropriate clusters, Algorithm 2 returns set *ssb*.
**Algorithm 2:** Clustering bags into sets of similar bags.**Input:***bags* – the list of the bags of words **Output:** list of clustered bags into sets of similar bags 1: **function** cluster_into_ssb(bags) 2: ssb = **new** set 3: unmatched_bags = bags.copy() 4: **while** (**not** unmatched_bags.empty()) **do** 5: seed = unmatched_bags.take_first() 6: ssb.add (create_similar_bags_list(seed, unmatched_bags)) 7: **end while** 8: **return** ssb 9: **end function**

**Algorithm 3:** Creating one set containing bags classified as similar.**Input**: *seed* – first bag of the list, *unmatched_bags*- container with uncompared bags **Output**: list of bags that are classified as similar 1: **function** create_similar_bags_list(seed, unmatched_bags) 2: similar_bags = **new** list 3: similar_bags.add(seed) 4: umatched_bags.remove(seed) 5: **foreach** (b in similar_bags) **do** 6: **foreach** (a **in** umatched_bags) **do** 7: **if** (similarity(b, a) > *eps_s*) 8: similar_bags.add(a) 9: unmatched_bags.remove(a) 10: **end if** 11: **end foreach** 12: **end foreach** 13: **return** similar_bags 14: **end function**

Algorithm 4 creates sequences from the list of similar bags of words. For this purpose, the considered list is sorted in ascending order according to the timestamps of bags of words. Subsequent bags of words are processed in *foreach* loop (line 6). The created object, *current_sequence*, represents the currently created sequence. The sequence is composed of subsequent bags of words within the time interval equal to or lower than *eps_time.* Subsequent bags of words are compared; in the case of timestamp difference (between the last and the current bag of words) higher than *eps_time*, the currently created sequence is terminated and added to the object *sequences* (line 8). Moreover, the time interval causing sequence termination is added to this sequence (*add_interval—*line 10; the term *current_sequence*[0]. *timestamp* denotes the timestamp of the first element of the *sequence* object). In the other case, the processed bag of words is added to *current_sequences*. Finally, operation *calculate_satistics_from_intervals()* provides statistical parameters (average, median, standard deviation—line 16) of the created sequences using time intervals derived during their creation.
**Algorithm 4:** Divides a similar bags list into sequences of bags.**Input:** *sbags* - the list of similar bags **Output:** sequences object that contains bag sequences list with interval time between successive sequences 1: **function** create_sequences (*sbags*) 2: sequences = **new** *sequences* 3: sbags.sort() 4: current_sequence = **new** list 5: last = sbags.first_bag() 6: **foreach** (b **in** s_bags) **do** 7: **if** (b.timestamp – last.timestamp > *eps_time*) 8: sequences.add(current_sequence) 9: sequences.add_timeRange(current_sequence[0].timestamp, last.timestamp) 10: sequences.add_interval(b.timestamp – current_sequence[0].timestamp) 11: current_sequence = **new** list 12: **end if** 13: current_sequence.add(b) 14: last = b 15: **end foreach** 16: sequences.calculate_satistics_from_intervals() 17: **return** sequences 18: **end function**

Algorithm 5 describes the method of *candidates’ creation based on the object sequences and ts_intervals. In the first step, objects* qualified as noise (Section 3) are filtered out from lists of sequences. This qualification is carried out in line 3 using the parameters *noise_0* and *noise_1* (compare Section 3). The noise relates to single periodical sequences. Such periodical logs do not indicate any anomaly or a single event that is pointed out by an interval object. Moreover, matching periodical logs leads to the generation of multiple offset values that could match OI. An infinite periodical signal shifted by a period is equal to the base signal. The algorithm drops noise sequences because they do not allow matching to the OI. In the *while* loop (line 7), the sequence matches are tested for each offset within the range*: < sequences.first_sequence().timestamp (i.e., initial timestamp of the sequence object), sequences.last_sequence().timestamp (i.e., last timestamp of the sequence object)>*. The offset value is incremented by *time_increment.* For each offset value procedure, *create_candidate* is invoked (line 8). If it returns a result different from NONE, then the generated candidate is added to the list *candidates* (lines 9 and 10). Procedure *create_candidate* is specified in Algorithm 6. It generates a candidate for matching using *offset* and *sequence* objects. In the first step, a new *evs* object is created as a copy of the original *ts_intervals* but with appended offset value. This is performed with the *copy_and_add_time_offset* method (line 2) applied to *ts.objects*. In line 6, *foreach* loop checks matching of the single sequence with a single interval in *ts_intervals*. If for every interval (element of *ts_intervals*), it is possible to match at least one sequence from the object *sequences*, then such a sequence object is treated as a *candidate*. Matching is verified by checking the inclusion of time ranges of the considered sequence and interval. Time ranges comprising initial and final timestamps are represented by *timeRanges* objects. The inclusion relation is tested with the procedure *includes()* (line 7). Non-matched sequences are returned via the object *filtered_sequences* (line 9) using the *add_sequence(s)* method. In the case of unsuccessful matching, the algorithm returns the value NONE.
**Algorithm 5:** Creates candidates list from one sequences object instance.**Input**: *sequences*- the sequences object, *events*- the object container that contains event object (each event is defined by start and end timestamps). **Output**: a list of candidates. Each candidate contains offset value and a sequences instance 1: **function** candidates_from_one_sequences (sequences, events) 2: candidates = **new** list 3: **if** (abs(s.avg – s.med)/s.avg < *noise_0* **and** s.std/s.avg < *noise_1*) 4: **return** candidates 5: **end if** 6: offset = sequences.first_sequence().timestamp 7: **while** (offset < sequences.last_sequence().timestamp) **do** 8: matched_sequences = create_candidate(sequences, events, offset) 9: **if** (matched_sequences != NONE) 10: candidates.add(**new** candidate(offset, matched_sequences)) 11: **end if** 12: offset = offset + *time_increment* 13: **end while** 14: **return** candidates 15: **end function**

**Algorithm 6:** Creates a candidate for matching with TS object with a given offset value, basing on the timestamp ranges of the sequences.**Input:** *i_sequences*- the set of sequences object, *ts_objects*- the object container that contains events object (each event is defined by start and end timestamps), *offset*- the time value in seconds **Output**: sequences object with sequence items that match with *ets_intervals* 1: **function** create_candidate (*i_sequences, ts_objects, offset*) 2: evs = ts_objects.copy_and_add_time_offset(offset) 3: current_interval = evs[0] 4: last_match = false 5: filtered_sequences = **new** sequences 6: f**oreach** (s **in** i_sequences) **do** 7: **if** (current_event.timeRanges.includes(s.timeRanges)) 8: last_match = true 9: filtered_sequences.add_sequence(s) 10: **else** 11: **if** (last_match) 12: current_interval = current_interval.next() 13: last_match = false 14: **if** (current_interval != NONE) 15: **break** 16: **end if** 17: **end if** 18: **if** (current_interval == NONE **or** (current_interval == evs.last_interval **and** last_match)) 19: **return** filtered_sequences 20: **end if** 21: **end foreach** 22: **return** NONE 23: **end function**

Algorithm 7 takes as the input the list of candidates in the form <*offset, matched sequences*>. It searches for an offset value with tolerance *eps_tolerance* to maximise the number of sequence objects with intervals in *events*. For each candidate *c* from the list *candidates,* the algorithm creates a list of candidates, for which the offset is in the range *<offset c, offset c + eps_tolerance*>. The length of this list is used to verify whether the new list is longer than the previous maximum value (line 11). In the case of satisfying this condition, the maximum value is updated, and the considered list is stored as the best list of candidates (line 12). The derived maximal list is returned by the algorithm. It corresponds to the RES(SBS_x_,k) set defined in Section 3.
**Algorithm 7:** Finds the best offset value (the offset value that generates the greatest number of matching candidates).**Input:***candidates* - the list of candidate object (defined by an offset and sequences) **Output**: a list of candidates with similar offset value creates the best matching with events 1: **function** find_best_matching (*candidates*) 2: max_matched_candidates = **new** list 3: foreach (c **in** candidates) **do** 4: matched_candidates = **new** list 5: matched_candidates.add(c) 6: **foreach** (d **in** candidates) **do** 7: **if** (d.offset >= c.offset and d.offset < c.offset + eps_tolerance **and** d **is not in** matched_candidates) 8: matched_candidates.add(d) 9: end if 10: end foreach 11: **if** (matched_candidates.count() > max_matched_candidates.count()) 12: max_matched_candidates = matched_candidates 13: **end if** 14: **end foreach** 15: **return** max_matched_candidates 16: **end function**

The developed algorithms have been used in practical analysis of some embedded devices. Section 5 presents illustrative results which give insight into data models and facilitate comprehension of algorithm operations.

## 5. Practical Examples

**Figure 1:**
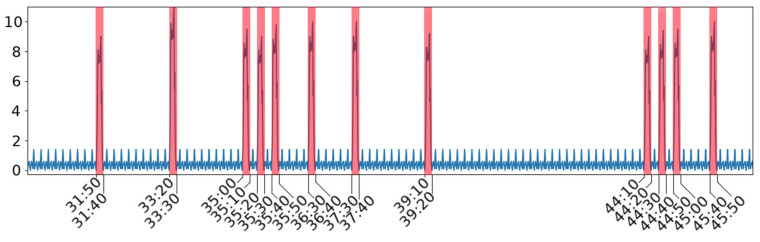
Example of time series for IoT device.

**Figure 2:**
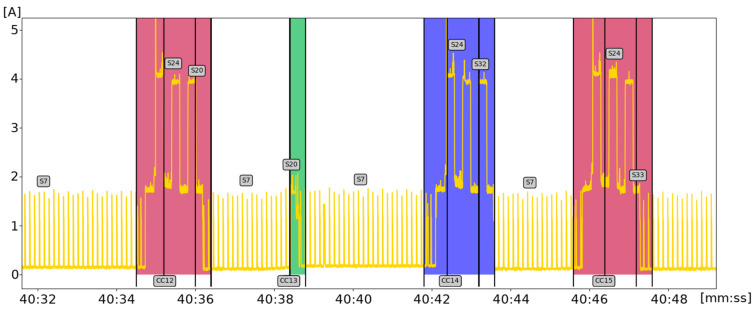
Excerpt of power supply plot for Holter device.

For better understanding of the proposed analysis and algorithms, we present practical examples. The developed methodology is targeted at embedded and IoT devices. Various operation features of such devices typically comprise some short periods of high activities on a background of less important processing or idle periods. This is illustrated in the time series of Figure 1 with 11 marked intervals (in red) corresponding to device high activity and background (considered as noise) in blue. The y-axis shows the percentage of CPU usage. The presented plot includes 1000 samples, and the x-axis corresponds to timestamps (minutes:seconds) which cover a period of 2000 s. A more detailed view of the plot needs a fine-grained time scale, and an excerpt of power consumption (in A) plot for the developed Holter device is presented in Figure 2. Here, three classes of time series intervals (red, blue, green) are presented; they relate to increased power consumption (current value) and can be interpreted by correlating them with matched event logs. Time series patterns denoted with red colour relate to two instances of the same TS object.

The event correlation process is illustrated for an excerpt of event logs presented in Table 1. We deal with a set of event logs listed in the first column of Table 1 (timestamps presented in <> brackets). In the first step of Algorithm 1, each element of event logs is transformed into a bag of words, e.g., log 1 “<2> [PER] checking it” results in the bag of words: [2, *timestamp*], [PER, *source*], [checking, *l-word*], [it, *l-word*]. Algorithm 2 combines logs into sets of similar bags of words. This algorithm uses the function *create_similar_bags_list ()* specified in Algorithm 3. The lists of created similar bags of words are given in the second column of the Table 1. For simplicity, in this table, we skip specification of word classes, and log sources are denoted in capital letters: PER—performance checking, DHCP—Dynamic Host Communication Protocol, and MANAGER—task manager.

Assuming parameter *eps_time* = 7 in Algorithm 4, we create object *sequences* from each list, which comprises single sequences denoted as sequence_0, sequence_2, etc. The created three objects of sequences (sequences1, sequences2, and sequences3) are specified as follows:

for list 1-> object sequences1:

sequence_0 = {<2> [PER] checking it}
interval_0 = 8
sequence_1 = {<10> [PER] checking it}
interval_1 = 8
sequence_2 = {<18> [PER] checking it}
interval_2 = 8
sequence_3 = {<26> [PER] checking it}
interval_3 = 8
sequence_4 = {<34> [PER] checking it}
interval_4 = 8
sequence_5 = {<42> [PER] checking it}
interval_5 = 8
sequence_6 = {<50> [PER] checking it}
interval_6 = 8
sequence_7 = {<58> [PER] checking it}

For list 2-> object sequences2:

sequence_0 = {<5> [DHCP] activity one, <6> [DHCP] activity two}
interval_0 = 20
sequence_1 = {<25> [DHCP] activity three}
interval_1 = 26
sequence_2 = {<51> [DHCP] activity four,<53> [DHCP] activity five}
For list 3-> object sequences3:
sequence_0 = {<9> [MANAGER] task one}
interval_0 = 14
sequence_1 = {<23> [MANAGER] task two, <24> [MANAGER] task three,
<27> [MANAGER] task four}
interval_1 = 29
sequence_3 = {<52> [MANAGER] task five, <54> [MANAGER] task six}

Most derived sequences comprise single bags of words; three involve two bags of words (lists 2 and 3) and one involves three bags of words (list 3). For example, bags of words “<2> [PER] checking it” and “<10> [PER] checking it” from list 1 create two separate sequences because the relevant timestamp difference is 8 s (>*eps_time* = 7). For list 3, the algorithm provides three sequences. The time difference between bags of words 4 and 7 is 14 s > *eps_time*. The difference between bags 7 and 8 is lower than the assumed *eps_time*. This results in two sequences: {<9> [MANAGER] task one} and {<23> [MANAGER] task two, <24> [MANAGER] task three, <27> [MANAGER] task four}.

For the created sequences, Algorithm 5 appends values of time differences (specified as *interval_x*) between sequences. These values are needed to calculate relevant median, average, and standard deviation metrics, which are used (Algorithm 7) to select a list of bags of words representing periodic (considered as noise in Section 3) and irregular log appearance. The latter most probably can match with the analysed time series objects. The standard deviation, the difference between median and average values for list 1, is equal to 0, which indicates periodical logs. Thus, this list is skipped in further processing (as opposed to Lists 2 and 3).

**Figure 3:**
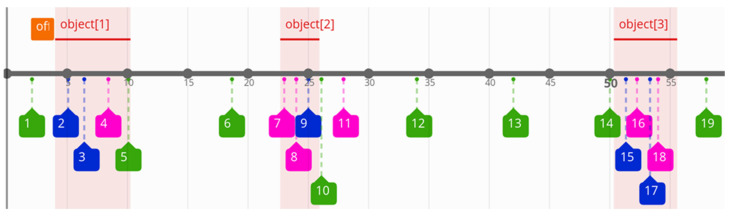
Matching event logs with time series objects.

Having processed event logs, we have to correlate them with relevant time series. In the considered time series (TS), we consider three object instances specified by time intervals (pairs of timestamps): {<7,13>, <25,28>, <53,58>}. Further processing needs timestamp normalisation in bags of words and TS intervals in reference to the first elements. For each bag of words, we have to subtract value 2 (the first timestamp in bags of words—compare Table 1) from subsequent timestamps. A similar operation for TS objects results in normalised intervals: {<0,7>, <18,21>, <46, 52>}. In the subsequent iterative steps (Algorithm 6), candidate objects are created for selected lists 2 and 3. The offset is the value which is added to each object’s timestamp during verification of the matching process. In this way, Algorithm 7 generates at least two candidates: <offset = 4, list_2> and <offset = 4, list_3>. Candidates with offset 4 are the unique ones with possible matching, which match the maximal number of candidates to TS objects. The result of matching is presented in Figure 3; here, the normalised time scale is replaced by the original one. Events related to lists 1, 2, and 3 are marked in green, blue, and red colours, respectively. The coloured boxes comprise the identification numbers of the relevant log records from the first column of Table 1. The highlighted objects (1–3) show the correlated events. It can be noticed that list_1 (green) is a periodic event and most of its instances are not correlated with the considered object occurrences. On the other hand, almost all elements of list_2 (blue) and list_3 (red) can be matched with the objects (taking into account the offset value equal to 2). In Figure 3, the orange box presents the offset value (relative to the timestamp of the first log record, this value is equal to 2 s). The offset value generates the first best matching result.

Optimizing power consumption of developed commercial Holter devices, we analysed time series related to the battery power supply current covering a longer period of typical device operation. It was provided by KeysigtData Acquisition Instrument (external monitoring with 2 ms sampling period defined by the local clock). Within the collected time series (covering time period of 2 h and 30 min), we identified several object instances (specified by time intervals—compare OI in Section 3) pointing to excessive power consumption device behaviour. The registered event logs comprised about 20,000 records. Time series intervals were identified using algorithms from our previous publication [2]. These intervals have been correlated with the collected event logs within the monitored Holter device (referred to its local clock). For this purpose, we used Algorithms 1–7 from Section 3. For an analysed TS sequence composed of three intervals—117 s, 120 s, and 119 s separated by 22 min 25 s and 27 min and 21 s, respectively—we found only 11 sets of similar bags of words that matched with these intervals. The presented approach is quite effective in selecting the correlated events from a bulk of recorded ones. In the analysed example, 11 event classes (sets of similar bags of words) contained almost 1600 log records in total. Most of them repeated in the consecutive three intervals, resulting in about 550 diverse records per interval; in fact, they constituted 550/11 = 50 event classes, which needed further investigation. They were generated by six software modules (specified as *source* type in the event record). Analysing these modules, we identified some deficiency in one of them, which caused power problems and needed correction. The correlation analysis significantly reduced the number of event logs needed for interpretation. This facilitated the tracing for problem sources in the monitored device.

## 6. Discussion

The main objective of the research was to develop efficient algorithms for correlating event and time series data collected during internal and external monitoring of embedded devices. The main issue was matching the time scales of two observation perspectives. This was based on iterative adjusting of time offsets to find the best matching of candidate event sequences with pointed time series intervals (Algorithms 6 and 7). These intervals are domain-dependent and can be defined manually or derived using time series decomposition algorithms, e.g., given in [2] or specified TS snippets, pulses, and other patterns. This issue is beyond the scope of the paper, since examples can be found in the literature (compare Section 2). The correlation scheme processes the event log sets, and, based on the introduced similarity metrics, derives potential events for matching. This results in adapting time scales defined by independent clocks of the monitored and monitoring devices. An important issue is that the matching process involves a sequence of instances related to a specified TS object. Moreover, the introduced event matching process filters out many events, so the interpretation of the correlation result is simpler (compare the Holter example in Section 5).

The developed algorithms use specified parameters. They should fit the features of the time series. It is assumed that the analysed time series include some repetitive activities with diverse distribution in time and low activity background. Such properties are quite typical for many signals characterising various operation properties of embedded and IoT devices. These properties impact selection of the algorithm parameters. They can be also refined experimentally for a given log format and characteristic class.

Monitoring device signals with independent data acquisition equipment (around its local clock) assures no impact on device operation, so the results are more accurate, and no hardware or software instrumentation are needed in the monitored device. This is in contrast to synchronised monitoring schemes that interact with the monitored device and can additionally limit the accuracy of monitoring fast processes.

The presented algorithms are consistent with data models specified in Section 3 and targeted at tracing correlations of time series object instances which appear in an aperiodic way. Single-instance and periodic objects were beyond our interest. However, some modifications of the algorithms could be introduced to handle such cases. In the conducted analysis, we assume that the investigated sequence of object instances corresponds to similar log records with similar time delays between the occurrence of the event and log record timestamps. In the case of high time delay jitter and low percentage of similar logs within subsequent object instances, the algorithm output may be not satisfactory. This can result from deficiencies in logging schemes (not correlated with the analysed objects) or lacking correlations. In the first case, some logging improvements can be considered.

The data analysis algorithms derive correlated events with pointed objects in time series. The effectiveness of this process depends to some extent on the assumed parameters related to the features of the object (duration, distance between subsequent object instantiations, noise factors) and their properties can be verified by checking the consistency of the result. Another issue is filtering logs that are not interesting, which depends on the used similarity function and the threshold parameter, leading to lower or higher levels of reduction of selected events. This can be trimmed by repeating algorithms for diverse threshold values and assessing results by the users (analytics). In the performed power consumption analysis of the Holter device, we correlated event logs correctly for the considered several object sequences; moreover, log reduction was quite significant.

The usefulness of the presented algorithms has been positively verified for some devices including developed commercial Holter devices. This allowed us to optimise device power consumption for longer operation times. The derived correlated logs facilitated pointing out deficiencies in hardware and software. Here, a question arises about the scope of application of the presented methodology. It is quite universal due to the object-oriented specifications. Time series intervals are specified in a natural way, and log event features can be easily adjusted, including other similarity metrics and noise specification.

## 7. Conclusions

The presented original algorithms extend the capabilities of analysing embedded and IoT device operation properties considering time series and event log repositories collected from internal and external monitoring processes. It is assumed that the time series study is targeted at specified time intervals (objects) pointed out by the investigator. For this purpose, other algorithms can be used, including those proposed in [2], or the experience within the device domain. The presented log processing aims at matching them with time series objects being investigated (typically comprising several or multiple instances). Here, an important issue is deriving time correlations between the monitoring and the monitored devices. This issue was neglected in the literature. The developed algorithms have been implemented and verified on real data. Their practical significance has been confirmed while developing commercial Holter devices.

The developed algorithms are specified in object-oriented pseudocode, which is quite natural for the time series and event log processing. Moreover, this facilitates introducing some modifications or extensions for better adaptation to diverse studied problems.

In future works, the following issues are worth investigating: (1) testing other similarity metrics and including event logs based on diverse log parsing patterns, and (2) verifying the impact of selecting parameters on algorithm results. Another interesting issue is to combine the introduced analysis with other time series decomposition and correlation schemes, e.g., involving deterministic, stochastic, seasonable, and trend components [3,18], and LSCWA and XWT approaches [16,17].

## Figures and Tables

**Table 1:** Excerpt of event logs.

Primary Event Logs	Lists of Similar Bags of Words
1: <2> [PER] checking it 2: <5> [DHCP] activity one 3: <6> [DHCP] activity two 4: <9> [MANAGER] task one 5: <10> [PER] checking it 6: <18> [PER] checking it 7: <23> [MANAGER] task two 8: <24> [MANAGER] task three 9: <25> [DHCP] activity three 10: <26> [PER] checking it 11: <27> [MANAGER] task four 12: <34> [PER] checking it 13: <42> [PER] checking it 14: <50> [PER] checking it 15: <51> [DHCP] activity four 16: <52> [MANAGER] task five 17: <53> [DHCP] activity five 18: <54> [MANAGER] task six 19: <58> [PER] checking it	List 1 <2> [PER] checking it <10> [PER] checking it <18> [PER] checking it <26> [PER] checking it <34> [PER] checking it <42> [PER] checking it <50> [PER] checking it <58> [PER] checking it List 2 <5> [DHCP] activity one <6> [DHCP] activity two <25> [DHCP] activity three <51> [DHCP] activity four <53> [DHCP] activity five List 3 <9> [MANAGER] task one <23> [MANAGER] task two <24> [MANAGER] task three <27> [MANAGER] task four <52> [MANAGER] task five <54> [MANAGER] task six

## Appendix A

In Table A1, we list the most important acronyms which were defined in Section 3. They facilitate tracing the presented considerations and algorithms.

**Table A1:** Basic acronyms used in the paper.

Acronym	Description
LSCWA	Least-Squares Cross-Wavelet Analysis
XWT	Cross-Wavelet Transform
TS	Set of time series samples
EL	Set of event log records
*OI*	Set of specified object intervals in time series
*SBSx*	Similar bags of words
*RES*(*SBS_x_*, *k*)	Set of event sequences within SBS_x_ set
*eps_s*,	Minimal similarity threshold
*eps_time*,	Parameter partitioning set SBSx into sequences
*noise_0*	Noise discrimination parameter for median
*noise_1*	Noise discrimination parameter for standard deviation
*time_increment*,	Parameter for matching event candidates (Algorithm 5)
eps_time_tolerance	Parameter for time offset tolerance (Algorithm 7)
